# SGLT2 Inhibitor Therapy and Long-Term Outcomes After Transcatheter Aortic Valve Implantation in Patients with Low Ejection Fraction

**DOI:** 10.3390/medicina62030535

**Published:** 2026-03-13

**Authors:** Berhan Keskin, Aykun Hakgor, Yerkenur Khidolda, Atakan Dursun, Aysel Akhundova, Umeyir Savur, Fatih Erkam Olgun, Ozlem Onder, Yasar Gokhan Gul, Beytullah Cakal, Bulent Demir, Haci Murat Gunes, Ibrahim Oguz Karaca, Ekrem Guler, Bilal Boztosun

**Affiliations:** 1 Department of Cardiology, Istanbul Medipol University, 34214 Istanbul, Türkiye; aykunhakgor@gmail.com (A.H.); atakandrsn@gmail.com (A.D.);; 2 Department of Anesthesiology, Istanbul Medipol University, 34214 Istanbul, Türkiye; yasar.gul@medipol.edu.tr

**Keywords:** transcatheter aortic valve implantation, SGLT2 inhibitors, mortality, cardiac remodeling, heart failure, low ejection fraction

## Abstract

*Background and Objectives*: Patients with impaired left ventricular ejection fraction (LVEF) undergoing transcatheter aortic valve implantation (TAVI) remain at high risk for adverse outcomes despite successful procedures. Sodium–glucose cotransporter-2 inhibitors (SGLT2i) improve outcomes in heart failure, but their long-term impact after TAVI is not well established. *Materials and Methods*: This single-center retrospective study included patients with LVEF < 50% who underwent transfemoral TAVI between January 2015 and September 2025. Patients were stratified according to SGLT2i use. The primary outcome was a composite of all-cause mortality and heart failure (HF) hospitalization requiring intravenous diuretics. Secondary outcomes included all-cause mortality, HF hospitalization, and changes in echocardiographic parameters at 6 months. Inverse probability of treatment weighting (IPTW) based on propensity scores was applied to adjust for baseline differences. Time-to-event analyses were performed using IPTW-weighted Cox models and adjusted survival curves. *Results*: The study included 226 patients (78 SGLT2i users, 148 non-users) with a median follow-up of 37 months. After IPTW adjustment, SGLT2i use was associated with a lower rate of the composite outcome (32.8% vs. 50.8%, *p* = 0.019) and a lower crude long-term mortality (32.8% vs. 47.4%, *p* = 0.056). Acute kidney injury after TAVI occurred less frequently among SGLT2i users (3.4% vs. 17.4%, *p* = 0.013). In IPTW-weighted Cox analyses, SGLT2i use was associated with a reduced risk of all-cause mortality (HR 0.57, 95% CI 0.32–0.98) and the composite outcome (HR 0.56, 95% CI 0.33–0.96). SGLT2i users demonstrated greater reductions in left ventricular end-diastolic diameter at 6 months. *Conclusions*: In patients with impaired LVEF undergoing TAVI, SGLT2 inhibitor therapy was associated with improved long-term survival, better composite outcome-free survival, and lower rates of post-TAVI acute kidney injury. Larger prospective studies are warranted to confirm these findings.

## 1. Introduction

The transcatheter aortic valve implantation (TAVI) population typically comprises patients with multiple comorbidities and an inherently high risk of subsequent adverse events. Impaired left ventricular ejection fraction (LVEF) is an established marker of higher risk for follow-up mortality and recurrent hospitalizations in patients undergoing TAVI, even after a technically successful procedure [1]. Among TAVI patients with impaired ejection fraction (LVEF < 50%), nearly half continue to exhibit persistently impaired LVEF during follow-up despite successful valve implantation [1]. Limited recovery of LVEF after TAVI has been associated with increased mortality and heart failure-related hospitalizations [1].

Landmark trials have demonstrated the beneficial effects of sodium–glucose cotransporter-2 inhibitors (SGLT2i) in patients with heart failure, particularly those with impaired ejection fraction, with significant reductions in heart failure hospitalizations and long-term mortality [2,3,4,5]. These benefits are thought to arise from multiple mechanisms, including anti-inflammatory, antioxidative, diuretic, metabolic, and renoprotective effects. Importantly, the reductions in mortality and hospitalization observed with SGLT2i appear to be largely independent of diabetes status or the severity of chronic kidney disease [2,3,4,5].

Despite the well-established benefits of SGLT2i in heart failure populations, their impact on long-term outcomes in patients with low LVEF undergoing TAVI remains insufficiently studied. In addition to potential prognostic benefits, SGLT2i may also facilitate favorable cardiac remodeling after TAVI. Therefore, this study aimed to evaluate the long-term effects of SGLT2i therapy on cardiac function and clinical outcomes, including all-cause mortality and heart failure hospitalizations, in patients with low LVEF undergoing TAVI.

## 2. Materials and Methods

### 2.1. Study Population

This single-center study included patients with LVEF <50% who underwent transfemoral TAVI for severe aortic stenosis between January 2015 and September 2025.

Exclusion criteria were as follows:

• In-hospital mortality (*n* = 21)
• Surgical conversion due to major structural complications (e.g., aortic dissection, device embolization, or annular rupture) (*n* = 3)
• End-stage renal disease (estimated glomerular filtration rate < 30 mL/min/1.73 m^2^ or chronic hemodialysis) (*n* = 12)
• Missing data (*n* = 23)
• Severe anemia (hemoglobin < 8 mg/dL) (*n* = 4)
• Valve-in-valve TAVI (*n* = 10)
• Active malignancy (*n* = 2)

**Figure 1:**
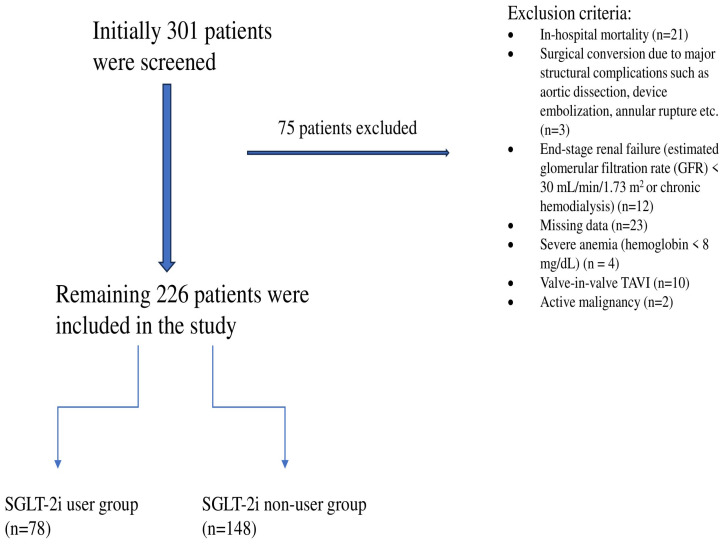
Study flow diagram.

After applying these exclusion criteria, 226 patients constituted the final study cohort (Figure 1).

The study was conducted in accordance with the ethical principles of the Declaration of Helsinki and was approved by the institutional ethics committee on 13 November 2025 (approval number: 1384). Written informed consent was obtained from all participants at hospital admission prior to invasive procedures, including consent for the scientific use of their data.

### 2.2. Data Collection and Procedural Information

Data on baseline clinical characteristics, laboratory parameters, medications, comorbidities, and procedural details at admission were obtained from the hospital electronic medical record system and patient files. SGLT2i therapy was continued during follow-up in patients who were already receiving these agents at the time of hospital admission. In addition, patients who were newly prescribed an SGLT2 inhibitor at hospital discharge (i.e., not receiving SGLT2i prior to hospitalization) initiated therapy during the index hospitalization and continued treatment throughout the follow-up period. All SGLT2i users received either empagliflozin 25 mg/day or dapagliflozin 10 mg/day. Information on hospitalization and long-term mortality was obtained from the hospital electronic database and the national health registry.

Transthoracic echocardiography was performed using a Vivid E95 system (GE Vingmed Ultrasound, Milwaukee, WI, USA) at hospital admission, with repeat evaluation at the 6-month post-procedural follow-up. All measurements were performed in accordance with the recommendations of the European Association of Cardiovascular Imaging and the American Society of Echocardiography [6]. LVEF was measured using the biplane Simpson’s method.

All patients underwent diagnostic coronary angiography via radial access, typically two days before the TAVI procedure. Decisions regarding revascularization were based on coronary anatomy and clinical presentation following a comprehensive case-based evaluation by the interventional heart team. In general, ≥90% stenosis in epicardial coronary arteries with a diameter ≥2.5 mm was considered hemodynamically significant and treated with percutaneous coronary intervention (PCI) when feasible.

All TAVI procedures were performed via transfemoral access under ultrasonographic and fluoroscopic guidance. A 6-Fr pigtail catheter was used for contrast injection during valve deployment. Temporary right ventricular pacing was established at the beginning of the procedure via the contralateral femoral vein. Pre-procedural computed tomography analyses were performed in all patients using 3mensio Structural Heart software (Pie Medical Imaging, Maastricht, The Netherlands) for valve selection and sizing. Vascular closure was achieved using Perclose ProGlide (Abbott Vascular, Santa Clara, CA, USA), with Angio-Seal (Terumo Corp., Tokyo, Japan) use when required. Surgical repair or covered stent implantation was performed in cases of major vascular complications or closure device failure.

### 2.3. Definitions and Outcomes

The primary outcome of the study was a composite of long-term all-cause mortality and hospitalization for heart failure (HF) requiring intravenous diuretic therapy. Secondary outcomes included all-cause mortality, HF hospitalization, and changes in LVEF and left ventricular dimensions on transthoracic echocardiography at 6-month post-procedural follow-up compared with baseline values.

Procedural outcomes, including structural, vascular, renal, and bleeding complications, were defined according to the Valve Academic Research Consortium-3 (VARC-3) criteria [7]. In accordance with VARC-3 definitions, bleeding events of type ≥2 were classified as major bleeding, whereas type 1 events were considered minor bleeding. Technical success and device success were also defined according to VARC-3 criteria.

### 2.4. Statistical Analysis

Patients were divided into two groups according to SGLT2i use. Categorical variables were compared using the chi-square test or Fisher’s exact test, as appropriate. Continuous variables were assessed for normality by visual inspection of histograms and the Kolmogorov–Smirnov test. Normally distributed variables were compared using Student’s *t*-test, whereas non-normally distributed variables were compared using the Mann–Whitney U test. Continuous data are presented as mean ± standard deviation for normally distributed variables and as median (interquartile range) for non-normally distributed variables. Categorical variables are reported as counts and percentages.

To minimize baseline differences between patients receiving SGLT2i and those not receiving these agents, inverse probability of treatment weighting (IPTW) based on propensity scores was applied. Propensity scores were estimated using a multivariable logistic regression model incorporating clinically relevant covariates, including age, sex, hypertension, diabetes mellitus, coronary artery disease, atrial fibrillation, baseline left ventricular ejection fraction, left ventricular end-diastolic diameter, pulmonary artery systolic pressure (PASP), valve type (self-expandable vs. balloon-expandable), and serum creatinine. Stabilized weights were calculated to improve precision and reduce the influence of extreme weights. In the weighted pseudo-population, baseline characteristics were summarized as weighted mean ± standard deviation for continuous variables and weighted percentages for categorical variables. Between-group comparisons were performed using weighted linear regression models for continuous variables and weighted generalized linear models with a binomial distribution for categorical variables. Covariate balance was assessed using standardized mean differences (SMD). Values <0.10 were considered indicative of good balance, whereas values between 0.10 and 0.20 were considered acceptable residual imbalance. Outcomes were compared after IPTW, and results are reported as weighted percentages.

SGLT2 inhibitor exposure was defined based on treatment use during the follow-up period for long-term analyses. All patients in the SGLT2i group received therapy throughout the entire follow-up period, with follow-up starting from the date of the TAVI procedure. In-hospital mortality events were excluded to minimize the influence of early procedural events on long-term outcomes.

To reduce potential bias arising from sparse risk sets at longer follow-up durations, a sensitivity time-to-event analysis was performed by administratively censoring follow-up at 5 years (60 months). IPTW based on propensity scores was applied to balance baseline covariates between SGLT2i users and non-users. Stabilized weights were incorporated into Cox proportional hazards models with robust variance estimation. IPTW-weighted Cox regression models were constructed to evaluate the association between SGLT2i use and outcomes, including the composite outcome and all-cause mortality. These models were additionally repeated using a 30-day landmark analysis to minimize the potential influence of early post-procedural events. Time-to-event outcomes were further assessed using IPTW-adjusted Kaplan–Meier survival curves. Between-group differences were explored using a weighted log-rank test; however, given the lack of a universally standardized weighted log-rank approach, primary statistical inference was based on IPTW-weighted Cox proportional hazards models with robust variance estimation.

Direct adjusted survival curves were generated using the Cox proportional hazards g-formula to further evaluate time-to-event outcomes. Adjusted survival probabilities were estimated under two hypothetical scenarios in which all patients were assumed to receive SGLT2 inhibitors or, alternatively, none received SGLT2 inhibitors, while maintaining their observed covariate profiles. This approach provides marginal survival estimates standardized to the overall study population. Survival probabilities were reported at prespecified time points (1, 3, and 5 years), and absolute risk differences were calculated accordingly. Restricted mean survival time (RMST) up to 60 months was computed to quantify average event-free survival time. Ninety-five percent confidence intervals for adjusted survival estimates and RMST were obtained using nonparametric bootstrap resampling with 500 iterations.

All statistical analyses were performed using Python version 3.14.0 (Python Software Foundation, Wilmington, DE, USA), and a two-sided *p*-value < 0.05 was considered statistically significant.

## 3. Results

The overall study cohort consisted of 226 patients, including 78 SGLT2i users and 148 non-users. In the unadjusted cohort, demographic characteristics were generally comparable between groups. However, SGLT2i users had a higher prevalence of diabetes mellitus (53.8% vs. 29.7%, *p* < 0.001), lower baseline LVEF (37.22 ± 8.99% vs. 41.96 ± 9.14%, *p* < 0.001), lower mean aortic gradient (39.11 ± 15.06 mmHg vs. 44.18 ± 15.00 mmHg, *p* = 0.019), and larger left-ventricular end-diastolic diameter (LVEDD) (5.43 ± 0.67 cm vs. 5.14 ± 0.69 cm, *p* = 0.004) and end-systolic diameter (LVESD) (4.05 ± 0.77 cm vs. 3.69 ± 0.84 cm, *p* = 0.003) (Table 1).

To reduce baseline confounding between SGLT2i users and non-users, inverse probability of treatment weighting (IPTW) based on propensity scores was applied. After IPTW adjustment, baseline clinical characteristics, comorbidities, laboratory values, medications, and echocardiographic parameters were generally well balanced between groups (Table 2). The median follow-up duration was 37.3 (14.7–54.6) months.

After IPTW adjustment, SGLT2i users demonstrated a significantly lower rate of the composite outcome compared with non-users (32.8% vs. 50.8%, *p* = 0.019). Similarly, long-term mortality occurred less frequently among SGLT2i users, with borderline statistical significance (32.8% vs. 47.4%, *p* = 0.056) (Table 3). Rates of heart failure hospitalization were comparable between groups (5.6% vs. 5.9%, *p* = 0.934). Procedural outcomes were generally similar, including device success (89.4% vs. 92.1%, *p* = 0.527) and technical success (92.6% vs. 97.6%, *p* = 0.115) (Table 3). The incidence of moderate-to-severe paravalvular leak did not differ significantly between groups (2.4% vs. 5.3%, *p* = 0.372). New permanent pacemaker implantation occurred more frequently among SGLT2i users (8.6% vs. 1.4%, *p* = 0.034), despite similar rates of self-expandable valve use (68.3% vs. 67.5%, *p* = 0.910) (Table 3). Acute kidney injury after TAVI was significantly less frequent among SGLT2i users (3.4% vs. 17.4%, *p* = 0.013), despite a higher contrast volume in the SGLT2i group (243.82 ± 140.32 mL vs. 201.72 ± 112.18 mL, *p* = 0.042), suggesting a potential nephroprotective effect of SGLT2i therapy (Table 3). Other periprocedural complications, including structural, vascular, and bleeding events, were comparable between groups (Table 3).

To further evaluate time-to-event outcomes, IPTW-weighted Cox regression analyses were performed. To minimize potential bias from sparse risk sets at extended follow-up, a sensitivity analysis with administrative censoring at 5 years (60 months) was conducted. The risk of the composite outcome was significantly lower among SGLT2i users (HR 0.56, 95% CI 0.33–0.96, *p* = 0.037) (Table 4). Similarly, SGLT2i use was associated with a significantly lower risk of long-term all-cause mortality compared with non-use (HR 0.57, 95% CI 0.32–0.98, *p* = 0.045) (Table 4). HF hospitalization was not significantly associated with SGLT2i therapy in IPTW-weighted Cox models.

In addition, IPTW-weighted Cox regression analyses were repeated using a 30-day landmark approach to better assess long-term outcomes after excluding early post-procedural events. This analysis also demonstrated more favorable outcomes in the SGLT2i group, including a lower risk of the composite outcome (HR 0.52, 95% CI 0.30–0.90, *p* = 0.020) and long-term mortality (HR 0.57, 95% CI 0.32–0.98, *p* = 0.045), whereas the risk of heart failure hospitalization did not differ significantly between groups (Table 4).

**Figure 2:**
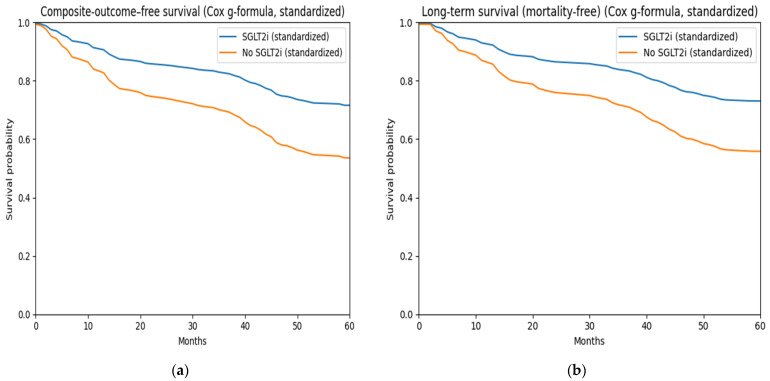
Direct adjusted survival curves. (**a**) Direct adjusted composite outcome-free survival; (**b**) direct adjusted overall survival.

**Figure 3:**
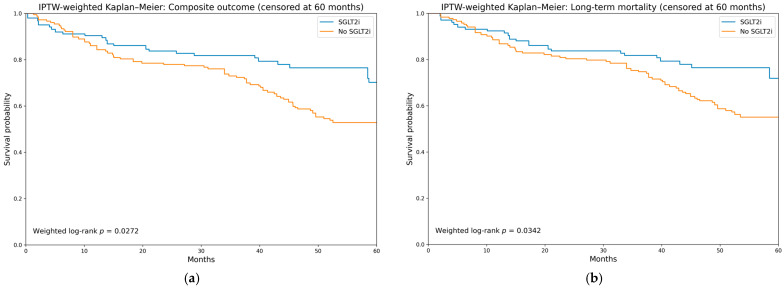
IPTW-weighted Kaplan–Meier curves. (**a**) IPTW-weighted Kaplan–Meier analysis for composite outcome-free survival; (**b**) IPTW-weighted Kaplan–Meier analysis for long-term all-cause mortality.

Direct adjusted survival curves generated using the Cox proportional hazards g-formula demonstrated consistently higher estimated survival probabilities among SGLT2i users throughout follow-up. Absolute survival differences were 3.8% at 1 year, 6.5% at 3 years, and 16.8% at 5 years (Table 4). A similar benefit was observed for composite outcome-free survival, with absolute differences of 4.3%, 8.9%, and 17.4% at 1-, 3-, and 5-year follow-up, respectively (Table 4). At 60 months, adjusted survival curves remained separated in favor of SGLT2i therapy (Figure 2). Restricted mean survival time (RMST) up to 60 months was 50.5 months for SGLT2i users and 45.9 months for non-users, corresponding to a mean survival gain of 4.6 months (95% CI −1.6 to 10.2) (Table 4). For composite outcome-free survival, the mean event-free survival gain was 5.6 months (95% CI: −0.8 to 11.8) for SGLT2i users (Table 4). IPTW-weighted Kaplan–Meier curves demonstrated a higher composite outcome-free survival probability (weighted log-rank *p* = 0.027) and improved overall survival (weighted log-rank *p* = 0.034) among SGLT2i users compared with non-users (Figure 3).

IPTW-weighted changes in echocardiographic parameters were compared between groups. Both groups demonstrated similar improvements in LVEF at follow-up (absolute change: 4.09 ± 6.98 vs. 4.64 ± 5.78, *p* = 0.618; relative change: 12.29 ± 22.52% vs. 12.74 ± 17.65%, *p* = 0.902 for SGLT2i users vs. non-users, respectively) (Table 5). LVEDD decreased significantly among SGLT2i users compared with non-users, with a greater absolute reduction (−0.13 ± 0.34 vs. −0.03 ± 0.17 cm, *p* = 0.040) and a greater percentage decrease (−2.08 ± 5.73% vs. −0.42 ± 3.16%, *p* = 0.048) (Table 5). In contrast, reductions in LVESD were comparable between groups (−0.15 ± 0.37 vs. −0.11 ± 0.41 cm, *p* = 0.617). Changes in PASP and mean aortic gradient were also similar between groups (Table 5).

## 4. Discussion

SGLT2i may confer clinical benefits in patients with impaired left ventricular ejection fraction undergoing TAVI through multiple mechanisms. Prior studies have demonstrated anti-inflammatory, antioxidative, and antifibrotic effects associated with SGLT2i therapy [8]. In addition, these agents have been shown to reduce myocardial stiffness, mitigate ventricular pressure overload, improve endothelial function, and enhance myocardial energetic efficiency [1,8,9]. A prior study by Mariani et al. [10] also demonstrated potential antiarrhythmic effects of SGLT2 inhibitors in patients with heart failure with reduced ejection fraction, particularly showing reductions in atrial fibrillation and ventricular tachycardia events. Given the established cardiovascular benefits and pleiotropic effects of SGLT2i and the known adverse prognostic impact of impaired ventricular systolic function in the TAVI population, these mechanisms may translate into meaningful clinical advantages. The present study provides long-term outcome data in this high-risk subgroup and evaluates potential associations with mortality and cardiac remodeling.

Evidence regarding long-term outcomes in this specific population remains limited. The only randomized trial to date, DAPA-TAVI [11], demonstrated lower rates of worsening heart failure and a reduction in the composite endpoint of death or worsening heart failure at 1-year follow-up among patients with heart failure undergoing TAVI. However, longer-term data are not yet available. Several observational studies have suggested potential benefits of SGLT2i in reducing long-term all-cause mortality and heart failure hospitalizations, although follow-up durations have generally been limited to approximately 2 years [12,13,14,15,16]. The present study provides longer-term follow-up, with a median duration exceeding 3 years and survival analyses extending to 5 years. Both crude mortality and the composite outcome were lower among SGLT2i users, and survival curves remained consistently separated in favor of SGLT2i throughout the 5-year follow-up period. In addition, IPTW-weighted Cox analyses demonstrated a statistically significant association between SGLT2i use and improved survival outcomes, including a lower risk of the composite outcome and long-term all-cause mortality.

SGLT2i use was not associated with a significant reduction in heart failure hospitalizations in our cohort; however, several factors may explain this finding. First, we adopted a relatively strict definition of HF hospitalization, including only admissions requiring intravenous diuretic therapy. This approach likely limited the number of events and reduced statistical power to detect between-group differences. Furthermore, hospitalizations culminating in death were analyzed separately as mortality events and were not counted as HF hospitalizations in order to avoid overlapping endpoints, which may have further lowered the observable hospitalization rate. The retrospective design also introduces the possibility of incomplete event capture or misclassification, particularly for admissions managed at external institutions with less detailed documentation. In contrast, prior randomized and observational studies have demonstrated reductions in HF-related hospitalizations with SGLT2i therapy, likely reflecting larger sample sizes, broader endpoint definitions, and prospective adjudication. Therefore, the absence of a significant difference in hospitalization in the present study should be interpreted cautiously and may reflect methodological and sample-size limitations rather than a true lack of effect. Larger prospective datasets and trials specifically powered for hospitalization outcomes are needed to better clarify this association.

The potential impact of SGLT2 inhibitors on cardiac remodeling after TAVI in patients with heart failure remains incompletely understood. Paolisso et al. [14] reported greater improvements in LVEF and left ventricular end-diastolic volume among SGLT2i users; however, the absence of matched baseline ventricular function parameters in that study raises the possibility of residual confounding. In our propensity-adjusted analysis, SGLT2i therapy was associated with a greater reduction in left ventricular end-diastolic diameter, whereas changes in LVEF and left ventricular end-systolic diameter were comparable between groups. As only one structural parameter demonstrated a differential change, these findings should be interpreted cautiously and do not allow definitive conclusions regarding a consistent remodeling benefit. Furthermore, the modest sample size and the relatively short echocardiographic follow-up of 6 months may have limited our ability to detect more subtle or delayed differences in ventricular functional recovery. Larger prospective studies with longer and more comprehensive imaging follow-up are warranted to better define the potential role of SGLT2i in post-TAVI ventricular remodeling.

Another key determinant of outcomes after TAVI is the development of acute kidney injury (AKI), which is associated with worse short- and long-term prognosis [17]. SGLT2i have demonstrated robust nephroprotective effects in major trials, independent of diabetes status and baseline renal function. Observational studies have also suggested a protective role in preventing contrast-associated AKI [18,19,20,21,22]. Paolisso et al. [23] previously reported a renoprotective effect of SGLT2i in the overall TAVI population. In the present study, SGLT2i use was associated with significantly lower rates of post-TAVI AKI among patients with low LVEF in IPTW-weighted analyses. Reduced AKI incidence and potential slowing of chronic kidney disease progression may represent an additional mechanism by which SGLT2i therapy contributes to improved long-term outcomes in this population.

Pleiotropic effects and cardiovascular benefits of SGLT2 inhibitors have also been demonstrated in patients with heart failure with preserved ejection fraction (HFpEF). Landmark randomized controlled trials have shown significant reductions in heart failure hospitalizations, composite cardiovascular outcomes, and improvements in quality-of-life measures with SGLT2i therapy in this population [3,4,24].

These benefits may likewise be relevant to TAVI patients with HFpEF. However, our study did not include patients with HFpEF; therefore, no conclusions can be drawn for this subgroup. Further prospective studies with larger sample sizes are needed to better clarify the potential role of SGLT2i therapy in this patient population.

## 5. Conclusions

In patients with low LVEF undergoing TAVI, SGLT2 inhibitor therapy was associated with lower long-term mortality, reduced composite adverse outcomes, and a lower incidence of post-procedural acute kidney injury, with consistently favorable survival trends observed up to 5 years. Larger prospective studies and randomized controlled trials are warranted to confirm these findings and to better define the role of SGLT2 inhibitors in the long-term management of patients with heart failure after TAVI.

## 6. Limitations

This study has several limitations. First, its retrospective, single-center design introduces the potential for selection bias, residual confounding, and limited generalizability. Although propensity score–based IPTW was applied to balance baseline characteristics, unmeasured confounders and treatment-selection bias cannot be fully excluded. Second, the sample size was modest, and event numbers were relatively low, particularly for heart failure hospitalizations, which may have limited statistical power to detect significant differences in time-to-event analyses. Third, SGLT2 inhibitor use was not randomized, and treatment initiation or continuation was at the discretion of treating physicians, raising the possibility of confounding by indication. Fourth, echocardiographic follow-up was limited to a 6-month assessment and may not have fully captured long-term ventricular remodeling after TAVI. Finally, systolic dysfunction was defined as LVEF < 50%, encompassing both heart failure with reduced ejection fraction and heart failure with mildly reduced ejection fraction; therefore, potential differences between guideline-defined EF subgroups could not be separately evaluated.

## Figures and Tables

**Table 1:** Comparison of baseline clinical characteristics, comorbidities, laboratory values, medications, and echocardiographic data in overall cohort.

Variable	SGLT2i Users (*n* = 78)	SGLT2i Non-Users (*n* = 148)	*p*-Value
**Baseline clinical characteristics**			
Age (years)	75.19 ± 9.51	76.76 ± 9.15	0.235
Body mass index (kg/m^2^)	27.11 ± 4.96	27.30 ± 4.37	0.773
Male sex	49 (62.8%)	84 (56.8%)	0.379
**Comorbidities**			
Hypertension	65 (83.3%)	128 (86.5%)	0.523
Diabetes mellitus	42 (53.8%)	44 (29.7%)	<0.001
Atrial fibrillation	33 (42.3%)	54 (36.5%)	0.393
COPD	42 (53.8%)	68 (45.9%)	0.259
Prior stroke	1 (1.3%)	11 (7.4%)	0.062
Chronic kidney disease	29 (37.2%)	56 (37.8%)	0.923
Coronary artery disease	50 (64.1%)	89 (60.1%)	0.560
Prior CABG	19 (24.4%)	26 (17.6%)	0.224
Preoperative pacemaker	2 (2.6%)	5 (3.4%)	1.000
**Laboratory values**			
Hemoglobin (g/dL)	11.59 ± 1.74	11.52 ± 1.70	0.774
Leucocytes (×10^9^/L)	7.79 ± 2.25	7.84 ± 2.76	0.878
Platelets (×10^9^/L)	219.85 ± 71.13	228.91 ± 81.16	0.388
Creatinine (mg/dL)	1.15 ± 0.41	1.17 ± 0.56	0.685
Albumin (g/dL)	4.20 ± 0.73	4.38 ± 0.76	0.172
Sodium (mmol/L)	137.17 ± 3.64	137.51 ± 3.79	0.507
AST (U/L)	41.02 ± 91.29	25.55 ± 16.88	0.153
TSH (mIU/L)	2.51 ± 3.06	1.97 ± 1.74	0.191
**Medications**			
ACEi/ARB	47 (60.3%)	88 (59.5%)	0.908
Beta-blocker	71 (91.0%)	128 (86.5%)	0.317
Statin	51 (65.4%)	82 (55.4%)	0.147
Insulin	16 (20.5%)	17 (11.5%)	0.068
Anticoagulation	30 (38.5%)	47 (31.8%)	0.312
**Echocardiographic parameters**			
LVEF (%)	37.22 ± 8.99	41.96 ± 9.14	<0.001
LVEDD (cm)	5.43 ± 0.67	5.14 ± 0.69	0.004
LVESD (cm)	4.05 ± 0.77	3.69 ± 0.84	0.003
Aortic Vmax (m/s)	3.78 ± 0.80	4.10 ± 0.64	0.003
Mean gradient (mmHg)	39.11 ± 15.06	44.18 ± 15.00	0.019
PASP (mmHg)	52.44 ± 13.89	54.20 ± 12.90	0.359
Low-flow low-gradient AS	24 (30.8%)	30 (20.3%)	0.078
Severe mitral regurgitation	21 (26.9%)	31 (20.9%)	0.396
Severe tricuspid regurgitation	19 (24.4%)	26 (17.6%)	0.298

Abbreviations: SGLT2i: Sodium–glucose cotransporter-2 inhibitor, COPD: Chronic obstructive pulmonary disease, CABG: Coronary artery bypass grafting, LVEF: Left ventricular ejection fraction, LVEDD: Left ventricular end-diastolic diameter, LVESD: Left ventricular end-systolic diameter, PASP: Pulmonary artery systolic pressure, AST: Aspartate aminotransferase, TSH: Thyroid-stimulating hormone, AS: Aortic stenosis.

**Table 2:** IPTW-weighted comparison of baseline clinical characteristics, comorbidities, laboratory values, medications, and echocardiographic data.

Variable	SGLT2i Users	Non-Users	*p*-Value (Weighted)	SMD After IPTW
**Baseline clinical characteristics**				
Age (years)	76.09 ± 8.95	76.30 ± 10.05	0.881	0.022
Body mass index (kg/m^2^)	27.36 ± 5.32	27.23 ± 4.45	0.867	0.026
Female sex	35.8%	40.1%	0.561	0.089
**Comorbidities**				
Hypertension	86.1%	88.4%	0.656	0.067
Diabetes mellitus	45.2%	39.7%	0.465	0.111
Atrial fibrillation	50.9%	43.4%	0.325	0.150
COPD	49.3%	47.8%	0.854	0.028
Prior stroke	1.0%	6.4%	0.094	0.282
Chronic kidney disease	39.9%	37.1%	0.704	0.058
Coronary artery disease	65.0%	62.0%	0.679	0.063
Prior CABG	28.0%	19.8%	0.199	0.193
Preoperative pacemaker	3.9%	3.6%	0.914	0.016
**Laboratory values**				
Hemoglobin (g/dL)	11.69 ± 1.79	11.57 ± 1.67	0.647	0.071
Leucocytes (×10^9^/L)	7.84 ± 2.34	7.93 ± 3.01	0.820	0.033
Platelets (×10^9^/L)	210.95 ± 68.51	222.74 ± 77.15	0.286	0.162
Creatinine (mg/dL)	1.16 ± 0.41	1.16 ± 0.55	0.991	0.001
Sodium (mmol/L)	137.11 ± 3.90	137.49 ± 3.81	0.523	0.099
AST (U/L)	22.00 [17.09–28.82]	20.96 [16.9–28.6]	0.687	0.223
TSH (mIU/L)	1.56 [0.93–2.34]	1.52 [0.92–2.78]	0.305	0.269
LDL-cholesterol (mg/dL)	125.07 ± 33.34	127.13 ± 45.52	0.769	0.052
Albumin (g/dL)	4.20 ± 0.74	4.31 ± 0.75	0.483	0.141
**Medications**				
ACEi/ARB	58.1%	63.4%	0.475	0.109
Beta-blocker	89.5%	89.9%	0.942	0.011
Statin	67.9%	54.5%	0.075	0.274
Insulin	16.1%	15.4%	0.897	0.019
Anticoagulation	47.7%	37.6%	0.180	0.204
**Echocardiographic data**				
LVEF (%)	39.69 ± 8.96	40.48 ± 9.45	0.578	0.085
LVDD (cm)	5.35 ± 0.65	5.30 ± 0.74	0.604	0.078
LVSD (cm)	3.94 ± 0.73	3.92 ± 0.93	0.885	0.021
Mean aortic gradient (mmHg)	40.52 ± 16.11	42.84 ± 15.48	0.352	0.147
PASP (mmHg)	52.90 ± 13.10	54.74 ± 12.90	0.360	0.142
Low-flow low-gradient AS	25.9%	26.4%	0.945	0.010
Severe mitral regurgitation	27.8%	22.0%	0.378	0.133
Severe tricuspid regurgitation	23.5%	15.8%	0.195	0.194

Abbreviations: SGLT2i: Sodium–glucose cotransporter-2 inhibitor, SMD: Standardized mean difference, IPTW: Inverse probability of treatment weighting, COPD: Chronic obstructive pulmonary disease, CABG: Coronary artery bypass grafting, LVEF: Left ventricular ejection fraction, LVEDD: Left ventricular end-diastolic diameter, LVESD: Left ventricular end-systolic diameter, LDL: Low-density lipoprotein, PASP: Pulmonary artery systolic pressure, AST: Aspartate aminotransferase, TSH: Thyroid-stimulating hormone, AS: Aortic stenosis.

**Table 3:** IPTW-weighted comparison of study outcomes and procedural data.

Variable	SGLT-2i Users	Non-Users	*p*-Value (Weighted)
**Study outcomes**			
Composite outcome	32.8%	50.8%	0.019
All-cause mortality	32.8%	47.4%	0.056
HF hospitalization	5.6%	5.9%	0.934
**Procedural data**			
Self-expandable valve use	68.3%	67.5%	0.910
Valve size (mm)	28.62 ± 3.91	29.09 ± 3.53	0.420
Total contrast volume (mL)	243.82 ± 140.32	201.72 ± 112.18	0.042
Device success	89.4%	92.1%	0.527
Technical success	92.6%	97.6%	0.115
Moderate-to-severe PVL	2.4%	5.3%	0.372
New permanent pacemaker	8.6%	1.4%	0.034
Coronary obstruction	1.0%	0.0%	0.999
Periprocedural stroke	0.0%	0.6%	0.999
Annular rupture	0.0%	0.0%	1.000
Aortic dissection	0.0%	0.0%	1.000
Cardiac tamponade	0.0%	0.6%	0.999
Device embolization	2.0%	2.3%	0.887
Acute kidney injury	3.4%	17.4%	0.013
Major vascular complication	15.0%	11.0%	0.420
Major bleeding	8.9%	9.4%	0.922

Abbreviations: SGLT2i: Sodium–glucose cotransporter-2 inhibitor, PVL: Paravalvular leak.

**Table 4:** IPTW-weighted time-to-event outcomes and survival benefit of SGLT2 inhibitors.

**Cox Proportional Hazards Analysis**				
Endpoint	Follow-Up (Months)	Hazard Ratio	95% CI	*p* -Value
Long-term mortality	60	0.567	0.325–0.988	0.045
Composite outcome	60	0.562	0.328–0.965	0.037
HF hospitalization	60	1.198	0.318–4.504	0.789
**Cox proportional hazards analysis with 30-day landmark**				
Endpoint	Follow-up (months)	Hazard ratio	95% CI	*p* -value
Long-term mortality	60	0.567	0.325–0.988	0.045
Composite outcome	60	0.523	0.303–0.904	0.020
HF hospitalization	60	0.772	0.184–3.236	0.723
**IPTW-adjusted survival probabilities (absolute risk at fixed time points)**				
**Long-term survival**				
Time-point	SGLT2i users	Non-users	Absolute difference (T1–T0)	
1-year	92.4%	88.6%	+3.8%	
3-year	81.8%	75.3%	+6.5%	
5-year	71.9%	55.1%	+16.8%	
**Composite outcome-free survival**				
Time-point	SGLT2i users	Non-users	Absolute difference (T1–T0)	
1-year	90.3%	86.0%	+4.3%	
3-year	81.8%	72.9%	+8.9%	
5-year	70.2%	52.8%	+17.4%	
**Restricted mean survival time (RMST) at 60 months (absolute time gained)**				
Endpoint	RMST (SGLT2i) (months)	RMST (Non-users) (months)	RMST difference (months gained)	95% CI
Long-term survival	50.5	45.9	+4.6	−1.6–10.2
Composite outcome-free survival	50.0	44.4	+5.6	−0.8–11.8

Abbreviations: CI: Confidence interval, HF: Heart failure, IPTW: Inverse probability of treatment weighting, SGLT2i: Sodium–glucose cotransporter-2 inhibitor.

**Table 5:** IPTW-weighted changes in echocardiographic parameters from baseline to 6 months follow-up.

Variables	Absolute Change (SGLT2i)	Absolute Change (Non-Users)	*p*-Value (Absolute Change)	% Change (SGLT-2i)	% Change (Non-Users)	*p*-Value (% Change)
LVEF (%)	4.09 ± 6.98	4.64 ± 5.78	0.618	12.29 ± 22.52	12.74 ± 17.65	0.902
LVEDD (cm)	−0.13 ± 0.34	−0.03 ± 0.17	0.040	−2.08 ± 5.73	−0.42 ± 3.16	0.048
LVESD (cm)	−0.15 ± 0.37	−0.11 ± 0.41	0.617	−2.87 ± 8.37	−2.07 ± 10.30	0.640
PASP (mmHg)	−13.78 ± 12.71	−15.27 ± 13.14	0.507	−24.80 ± 22.83	−24.58 ± 24.08	0.957
Mean aortic gradient (mmHg)	−32.43 ± 15.25	−34.24 ± 16.54	0.538	−76.82 ± 15.27	−75.98 ± 22.68	0.818

Abbreviations: SGLT2i: Sodium–glucose cotransporter-2 inhibitor, LVEF: Left ventricular ejection fraction, LVEDD: Left ventricular end-diastolic diameter, LVESD: Left ventricular end-systolic diameter, PASP: Pulmonary artery systolic pressure.

## Data Availability

Data supporting the results of this work can be obtained from the corresponding author upon reasonable request.

## Abbreviations

The following abbreviations are used in this manuscript:

LVEF	Left ventricular ejection fraction
TAVI	Transcatheter aortic valve implantation
SGLT2i	Sodium–glucose cotransporter-2 inhibitors
HF	Heart failure
IPTW	Inverse probability of treatment weighting
PCI	Percutaneous coronary intervention
VARC-3	Valve Academic Research Consortium-3
SMD	Standardized mean differences
RMST	Restricted mean survival time
LVEDD	Left-ventricular end-diastolic diameter
LVESD	Left-ventricular end-systolic diameter
PASP	Systolic pulmonary artery pressure
AKI	Acute kidney injury
